# Supplementary material for: Stretchable OLEDs based on a hidden active area for high fill factor and resolution compensation
Source: Nat Commun. 2024 Jun 4;15:4349. doi: 10.1038/s41467-024-48396-w (PMC11150391; doi:10.1038/s41467-024-48396-w)

Supplementary Information

Stretchable OLEDs based on a hidden active area for high fill factor and resolution compensation

Donggyun Lee[1], Su-Bon Kim[1], Taehyun Kim[1], Dongho Choi[1], Jee Hoon Sim[1], Woochan Lee[1], Hyunsu Cho[2], Jong-Heon Yang[2], Junho Kim[1], Sangin Hahn[1], Hanul Moon[3]★, and Seunghyup Yoo[1]★

Affiliations

[1] School of Electrical Engineering, Korea Advanced Institute of Science and technology (KAIST), Daejeon 34141, Republic of Korea

[2] Electronics Telecommunications Research Institute (ETRI), Daejeon 34129, Republic of Korea

[3] Department of Semiconductor; Department of Chemical Engineering (BK21 FOUR Graduate Program), Dong-A University, Busan 49315, Republic of Korea

★Correspondence and requests for materials should be addressed to H.M. (email:hmoon@dau.ac.kr) or S.Y. (email: syoo@ee.kaist.ac.kr).

The PDF file includes:

Supplementary Fig. 1 to Fig. 21

Other Supplementary Material for this manuscript includes the following:

Supplementary Movie 1 to 8

Supplementary Note 1 | The mathematical relationships of a fill factor as a function of system strain for both stretchable OLEDs with the conventional platform and the proposed hidden active area (HAA) platform.

A. Common expression

As mentioned in the main text, the system strain (ε_{sys}) can be represented as shown below:

$$\varepsilon_{\text{sys}} = \frac{\Delta L_{\text{cell}}}{L_{\text{cell}}} = \frac{\Delta L_{\text{int}}}{L_{\text{cell}}} = \frac{L'_{\text{int}} - L_{\text{int}}}{L_{\text{is}} + L_{\text{int}}} \qquad \text{(S1)}$$

in which, L_{is}, L_{int}, and L_{cell} are the lengths of a rigid island, a stretchable interconnector, and a unit cell that consists of the island and the interconnector. L_{int} and L_{cell} are the L'_{int} and L'_{cell} values when ε_{sys} is zero.

Rewriting **Equation (S1)** in terms of L_{int} yields **Equation (S2)**.

$$L'_{\text{int}} = L_{\text{int}} + \Delta L_{\text{cell}} = (\varepsilon_{\text{sys}} + 1)L_{\text{int}} + \varepsilon_{\text{sys}} L_{\text{is}} \qquad \text{(S2)}$$

B. Conventional case

Fill factors of an initial state ($FF_0^{(c)}$) and a stretched state ($FF^{(c)}$) are expressed by **Equation (S3)** and **(S4)**, respectively.

$$FF_0^{(c)} = \frac{A_{\text{is}}}{A_{\text{cell}}} = \left(\frac{L_{\text{is}}}{L_{\text{is}} + L_{\text{int}}}\right)^2 \qquad \text{(S3)} \qquad\qquad FF^{(c)} = \frac{A_{\text{is}}}{A'_{cell}} = \left(\frac{L_{\text{is}}}{L_{\text{is}} + L'_{\text{int}}}\right)^2 \qquad \text{(S4)}$$

$A_{\text{is}} = (L_{\text{is}})^2$, $A_{\text{cell}} = (L_{\text{is}} + L_{\text{int}})^2$ and $A'_{\text{cell}} = (L_{\text{is}} + L'_{\text{int}})^2$ are the areas of the rigid island and the unit cell. Subsequently, by substituting **Equations (S2)** and **(S3)** into **Equation (S4)**, the equation presented as **Equation (1)** in the manuscript can be derived as shown below.

$$FF^{(c)} = \left(\frac{L_{\text{is}}}{L_{\text{is}} + (\varepsilon_{\text{sys}} + 1)L_{\text{int}} + \varepsilon_{\text{sys}} L_{\text{is}}}\right)^2 = \left(\frac{L_{\text{is}}}{(\varepsilon_{\text{sys}} + 1)(L_{\text{is}} + L_{\text{int}})}\right)^2 = \frac{FF_0^{(c)}}{(\varepsilon_{\text{sys}} + 1)^2} \qquad \textbf{Equation (1)}$$

C. Proposed case

Fill factors of an initial state ($FF_0^{(p)}$) and a stretched state ($FF^{(p)}$) are expressed by **Equation (S5)** and **(S6)**, respectively.

$$FF_0^{(p)} = \frac{A_{\text{is}} + 2A_{\text{hidden}}}{A_{\text{cell}}} = 1 - \left(\frac{L_{\text{int}}}{L_{\text{is}} + L_{\text{int}}}\right)^2 \qquad \text{(S5)} \qquad\qquad FF^{(p)} = \frac{A_{\text{is}} + 2A'_{\text{hidden}}}{A'_{\text{cell}}} = 1 - \left(\frac{L'_{\text{int}}}{L_{\text{is}} + L'_{\text{int}}}\right)^2 \qquad \text{(S6)}$$

$A_{\text{hidden}} = L_{\text{is}} \times L_{\text{int}}$ and $A'_{\text{hidden}} = L_{\text{is}} \times L'_{\text{int}}$ is the area of HAA. By rearranging **Equation (S5)** for L_{is}, **Equation (S7)** shown below is derived.

$$L_{\text{is}} = \left(\frac{1}{\sqrt{1 - FF_0^{(\text{p})}}} - 1 \right) L_{\text{int}} \qquad \text{(S7)}$$

And by substituting **Equations (S7)** into **Equation (S2)**, **Equation (S8)** can be modified as shown below.

$$L'_{\text{int}} = \left(\frac{\varepsilon_{\text{sys}}}{\sqrt{1 - FF_0^{(\text{p})}}} + 1 \right) L_{\text{int}} \qquad \text{(S8)}$$

Consequently, by substituting **Equations (S7)** and **(S8)** into **(S6)**, **Equation (2)** in the manuscript can be derived.

$$FF^{(\text{p})} = \frac{FF_0^{(\text{p})}}{(\varepsilon_{\text{sys}}+1)^2} \left[1 + 2\varepsilon_{\text{sys}} \left(\frac{1 - \sqrt{1 - FF_0^{(\text{p})}}}{FF_0^{(\text{p})}} \right) \right] \qquad \textbf{Equation (2)}$$

Equation (1) and **(2)** can be more simplified in the range of very small ε_{sys}. Considering an arbitrary function $FF_{(\varepsilon)}$ and a point of $\varepsilon = \varepsilon_0$, $FF_{(\varepsilon_0 + d\varepsilon)}$ can be approximated by taking the first two terms of Taylor expansion.

$$FF_{(\varepsilon_0 + d\varepsilon)} = FF_{(\varepsilon_0)} + \frac{1}{1!} \frac{\partial FF_{(\varepsilon)}}{\partial \varepsilon_0}\bigg|_{\varepsilon=\varepsilon_0} d\varepsilon + \frac{1}{2!} \frac{\partial^2 FF_{(\varepsilon)}}{\partial {\varepsilon_0}^2}\bigg|_{\varepsilon=\varepsilon_0} d\varepsilon^2 + \frac{1}{3!} \frac{\partial^3 FF_{(\varepsilon)}}{\partial {\varepsilon_0}^3}\bigg|_{\varepsilon=\varepsilon_0} d\varepsilon^3 + \cdots \approx FF_{(\varepsilon_0)} + \frac{\partial FF_{(\varepsilon)}}{\partial \varepsilon_0}\bigg|_{\varepsilon=\varepsilon_0} d\varepsilon$$

If $\varepsilon_0 = 0$ and ε is very small. *i.e.* $\varepsilon \approx d\varepsilon \ll 1$,

$$FF_{(\varepsilon_0 + d\varepsilon)} = FF_{(\varepsilon)} = FF_{(0)} + \frac{\partial FF_{(\varepsilon)}}{\partial \varepsilon_0}\bigg|_{\varepsilon=0} \varepsilon \qquad \text{(S9)}$$

By applying **Equation (1)** and **Equation (2)** to **Equation (S9)**, simplified versions of $FF^{(\text{c})}$ and $FF^{(\text{p})}$ can be derived as shown below.

$$FF^{(\text{c})} = \frac{FF_0^{(\text{c})}}{(\varepsilon_{\text{sys}}+1)^2} \approx (1 - 2\varepsilon_{\text{sys}}) FF_0^{(\text{c})}, \text{ when } \varepsilon \ll 1 \qquad \textbf{Equation (1')}$$

$$FF^{(\text{p})} = \frac{FF_0^{(\text{p})}}{(\varepsilon_{\text{sys}}+1)^2} \left[1 + 2\varepsilon_{\text{sys}} \left(\frac{1 - \sqrt{1 - FF_0^{(\text{p})}}}{FF_0^{(\text{p})}} \right) \right] \approx (1 - 2\varepsilon_{\text{sys}}) FF_0^{(\text{p})} + 2\varepsilon_{\text{sys}} \left(1 - \sqrt{1 - FF_0^{(\text{p})}} \right), \text{ when } \varepsilon \ll 1 \qquad \textbf{Equation (2')}$$

D. Design objectives

The design objectives are to achieve a $FF_0^{(\mathrm{p})}$ higher than 95% and a $FF^{(\mathrm{p})}$ higher than 85% at a $\varepsilon_{\mathrm{sys}} = 30\%$. Since the OLED is deposited on a 40 mm by 40 mm substrate, the size of the OLED should be smaller than this. Therefore, the dimensions of the 6 × 6 islands were set to 1 inch (25.4 mm × 25.4 mm) for ease of deposition. When $L_{\mathrm{is}} =$ 2.9 mm, L'_{int} is determined as 1.6 mm, achieving a $FF^{(\mathrm{p})} =$ 87%, aligning with the objective. Meanwhile, based on these determined dimensions, to achieve a system strain of approximately 30%, L_{int} is calculated to be approximately 0.56 mm. Therefore, by setting L_{int} to 0.6 mm, the $FF_0^{(\mathrm{p})}$ aligns with the design objective at 97%. Furthermore, as evident from **Supplementary Equations (S1)** and **(S5)**, minimizing L_{int} maximizes system strain while increasing $FF_0^{(\mathrm{p})}$. However, reducing L_{int} to below 0.6 mm results in different parts of the HAA adhering to each other, potentially leading to the mechanical failure of the initial state HAA structure. Therefore, we optimized it to 0.6 mm, which is feasible in the fabrication process, ensures a stable structure, and maximizes the $FF_0^{(\mathrm{p})}$.

Supplementary Figure 1 | The geometry of the shadow mask used for OLED deposition and FIB-SEM image analysis. (a) Schematic diagrams of shadow masks to define the patterns of each layers in the proposed stretchable OLED device. (b) The top views of a deposited area through the shadow masks of each OLED layers. (c) A FIB-SEM cross sectional image of the fabricated OLED.

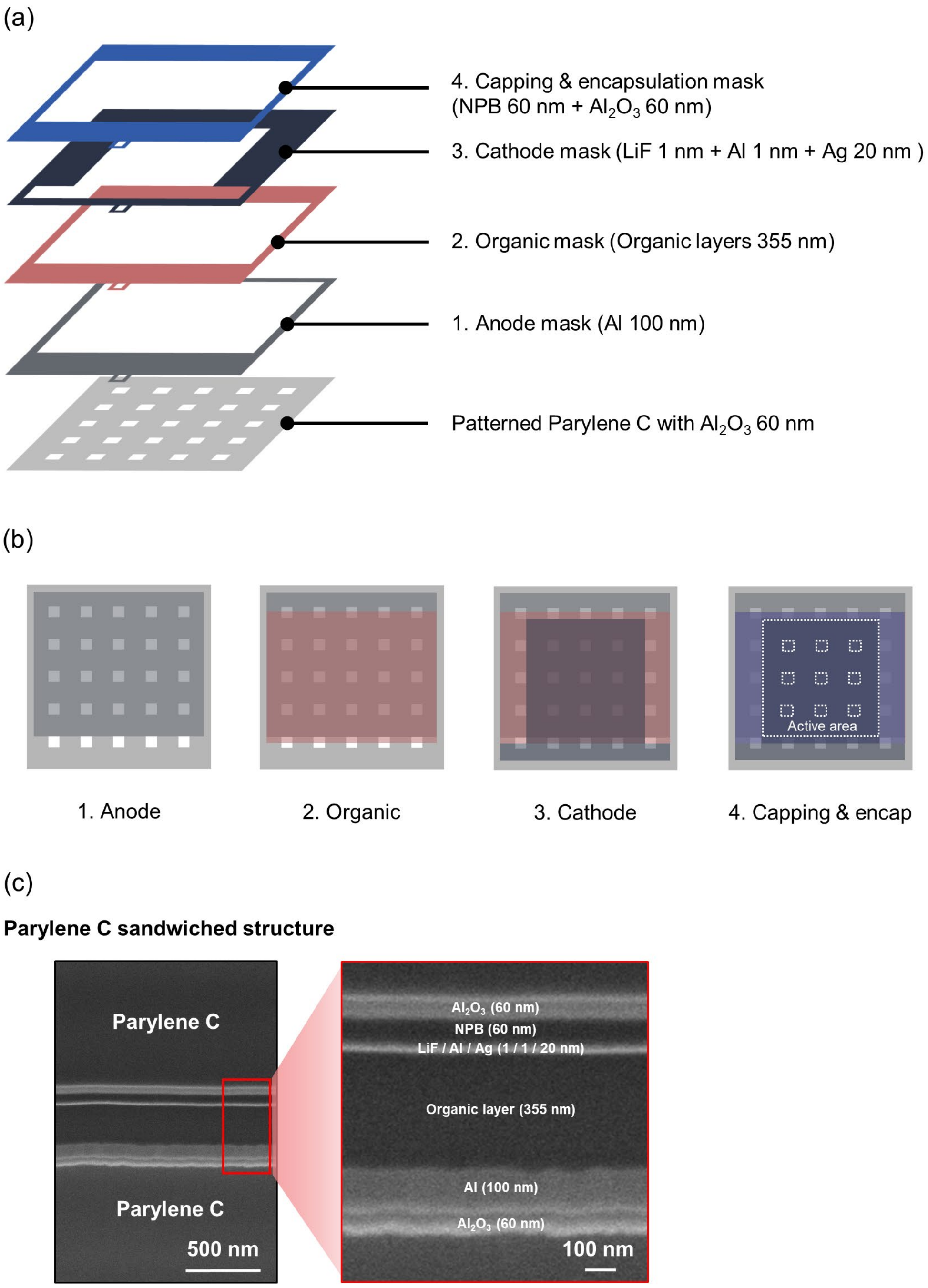

Supplementary Figure 2 | Photographs of each steps in the fabrication process of free-stranding ultrathin OLED. (a) A 4 cm by 4 cm carrier glass with fluorinated polymer (1 μm) / bottom Parylene C (1 μm) formed, and a shadow mask with square holes for patterning the bottom Parylene C. (b) The samples placed inside the RIE chamber. (c) A patterned bottom Parylene C substrate. (d) Formation of the bottom encapsulation layer (Al_2O_3) through atomic layer deposition (ALD). (e) A sample after the depositions of anode, organic layer, and cathode. (f) Formation of the top encapsulation layer (Al_2O_3) through the process of ALD. (g) A sandwiched OLED on a carrier glass after the formation of the top Parylene C (1 μm) layer, and a shadow mask for the square hole patterns and pad opening in the top Parylene C. (h) The OLED samples placed inside the RIE chamber. (i) A free-standing Parylene C sandwiched OLED device detached from the carrier glass.

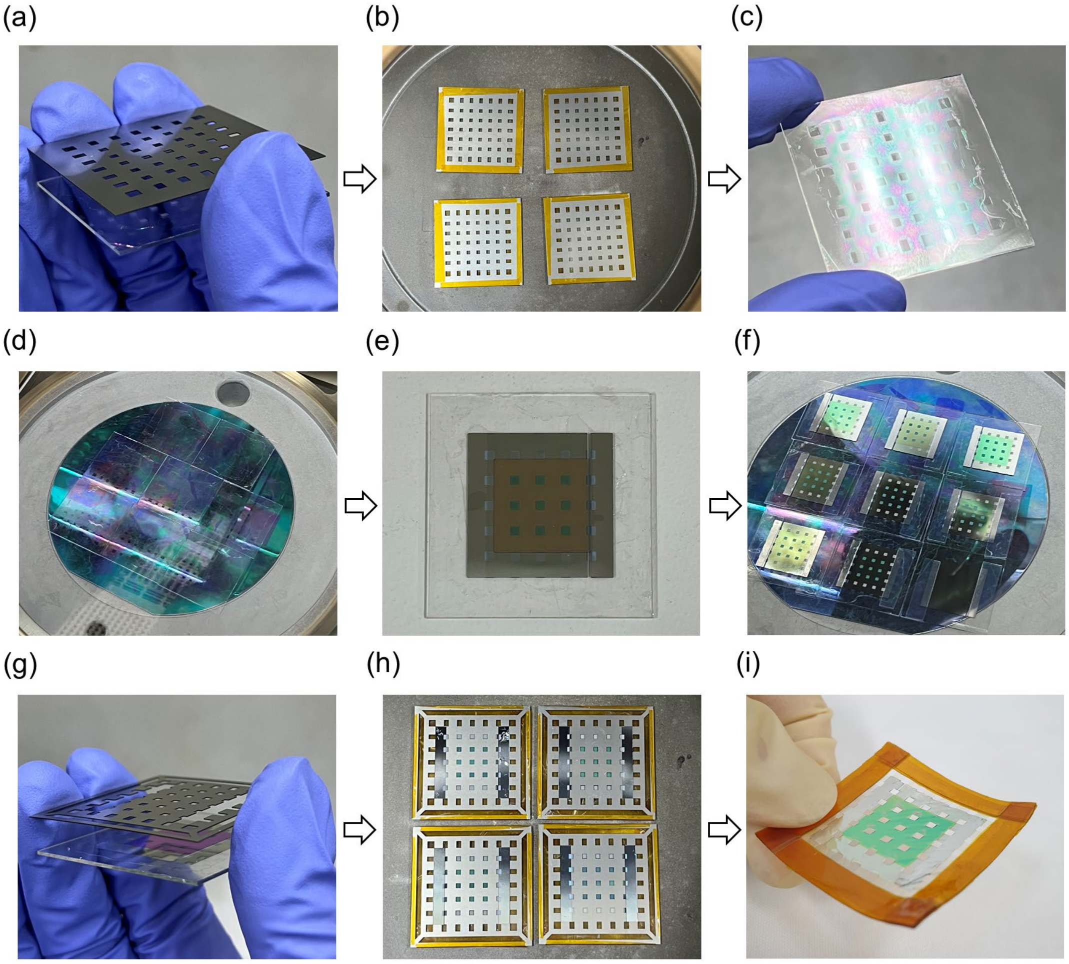

Supplementary Figure 3 | Photographs of each steps in the fabrication process of patterned-hybrid elastomer and transferring the ultrathin OLED onto it. (a) Pouring PDMS onto an Al mold with a 1mm-thick black spacer, and using a doctor blade to remove residual PDMS over the 6 × 6 arrays of square islands. (b) Pouring Ecoflex^{TM} 00-20 onto the Al mold with the PDMS island arrays and peeling it off from the mold after curing. (c) Transferring and adhering the ultrathin OLED onto the pre-stretched patterned-hybrid elastomer. (d) Releasing the pre-strain. (e) The stretchable OLED device driven at 1 mA after being removed from the stage.

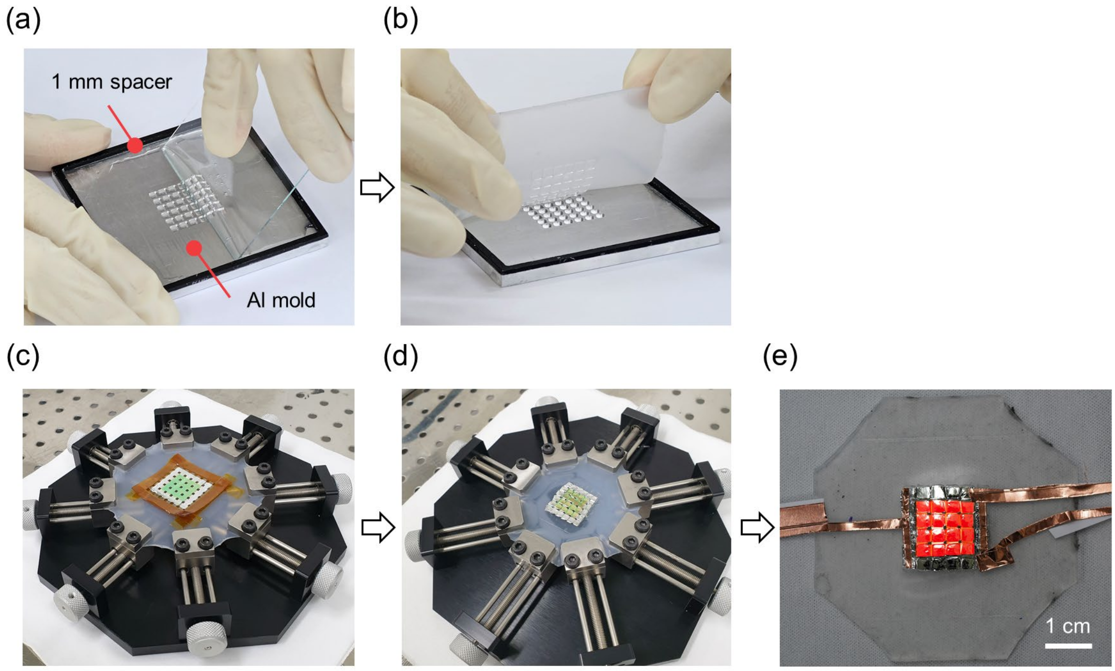

Supplementary Figure 4 | Detailed boundary conditions for the ANSYS simulation. (a) Tilt view and side view of the quadaxial stretching simulation. The surfaces to be grabbed to initiate and maintain displacement are highlighted in red, while PDMS and Ecoflex^{TM} are depicted in dark gray and light gray, respectively. (b) Tilt view of the orthogonal biaxial stretching. (c) Tile view of the diagonal biaxial stretching.

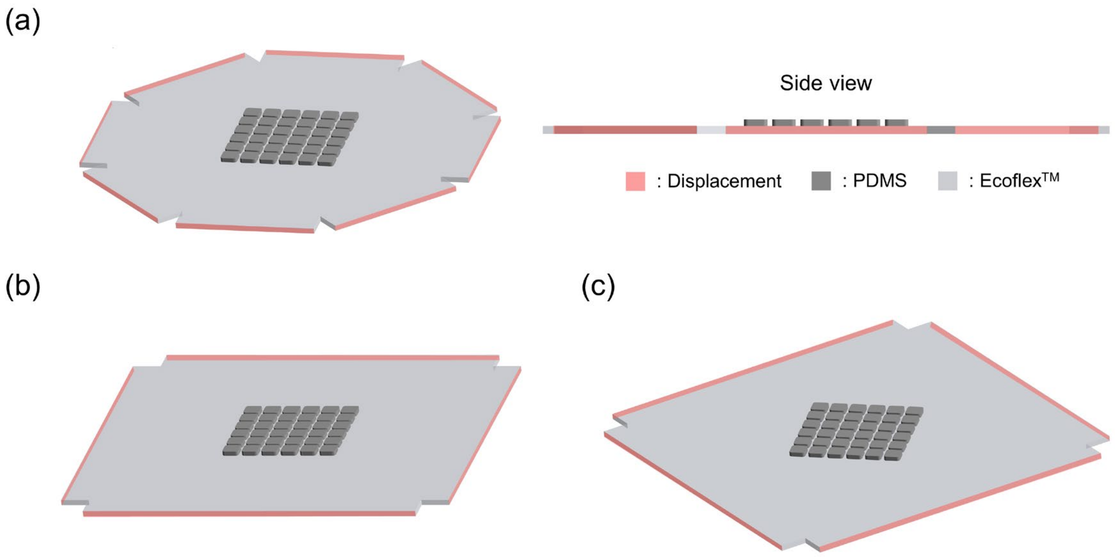

Supplementary Figure 5 | Analysis of alignment accuracy based on $\varepsilon_{diag}/\varepsilon_{ortho}$ ratios through ANSYS simulation. The ANSYS simulation results for deformation along the *y*-axis of the patterned-hybrid elastomer under stretching, at $\varepsilon_{diag}/\varepsilon_{ortho}$ ratios of (a) 0, (b) 0.5, and (c) 1. Plots in (d), (e), and (f) show the magnified view for the results of the *y*-axis deformation for the area of the 6 × 6 island arrays in each case.

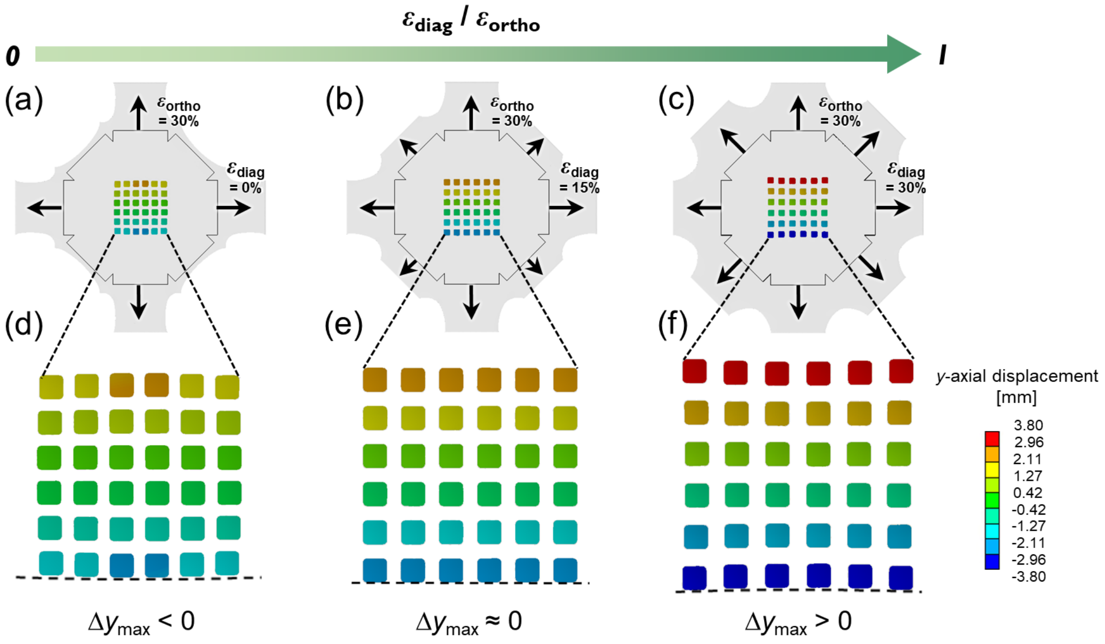

Supplementary Figure 6 | Analysis of alignment accuracy based on stretching methodology through ANSYS simulation. The ANSYS simulation results for y-axis deformation of the patterned-hybrid elastomer under stretching in (a) quadaxial, (b) orthogonal, and (c) diagonal directions. (d), (e), and (f) display the magnified results of the y-axis deformation for the area of the 6 × 6 island arrays in each case.

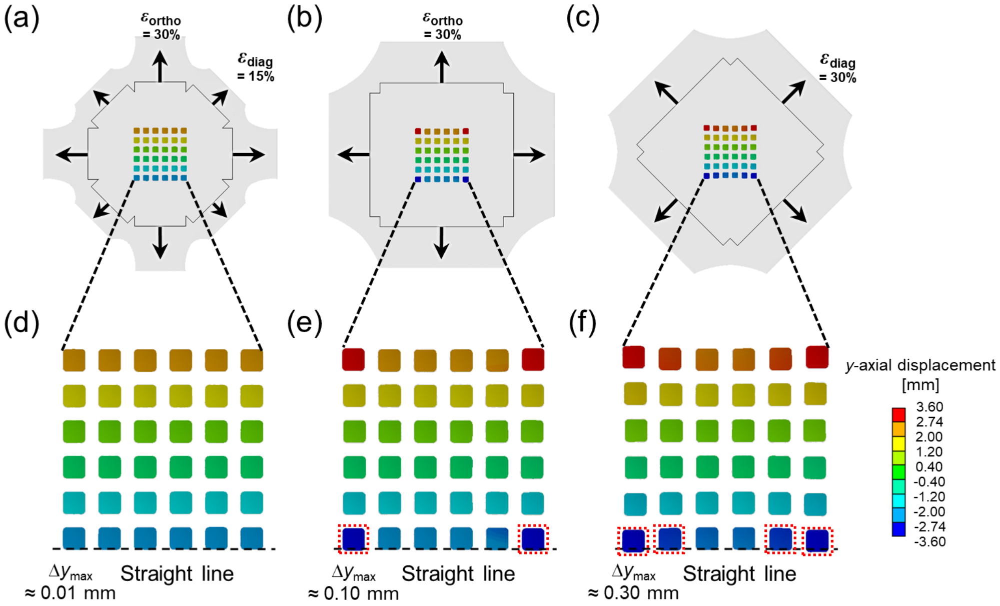

Supplementary Figure 7 | Detailed boundary conditions for the COMSOL simulation. The magnified view on the right shows the appearance of the thin-film OLED bonded to the PDMS.

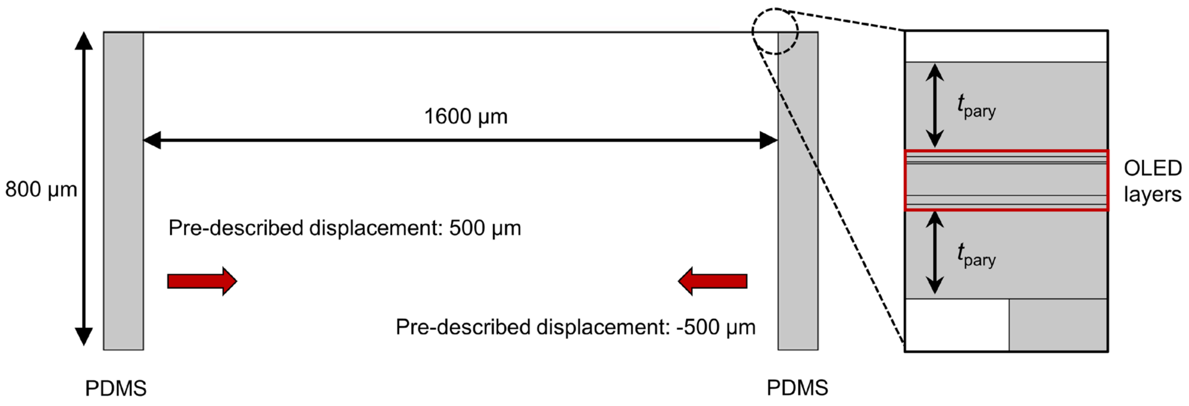

Supplementary Figure 8 | Simulated strain tensor along the *z*-axis at the position A in the *x*-axis which is depicted in Fig. 4 in the main manuscript. (a) *xx* strain tensor, (b) *zz* strain tensor, and (c) *zx* strain tensor according to the thickness of Parylene C film (t_{pary}).

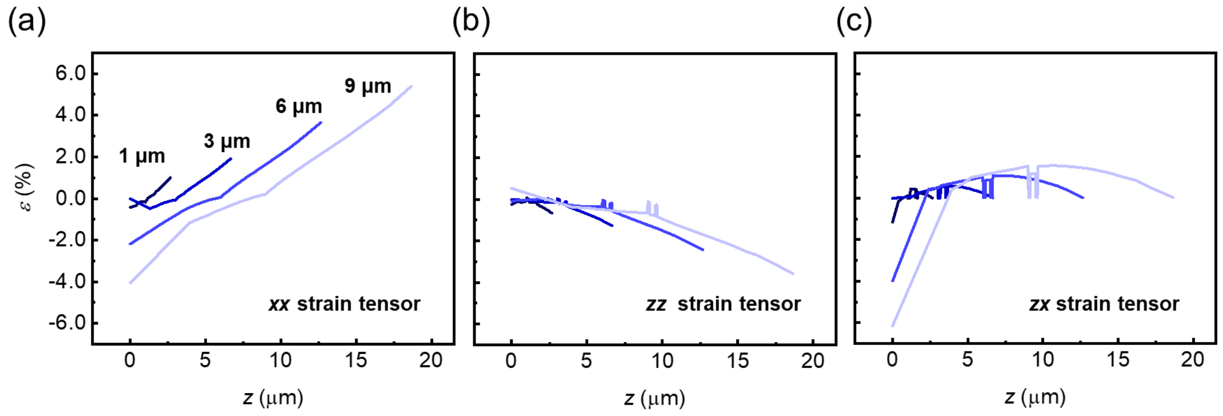

Supplementary Figure 9 | The current density and luminance characteristics versus driving voltage up to 5.25V for the different biaxial system strain (ε_{sys}) values, measured in order of 30%, 20%, 10%, and 0%.

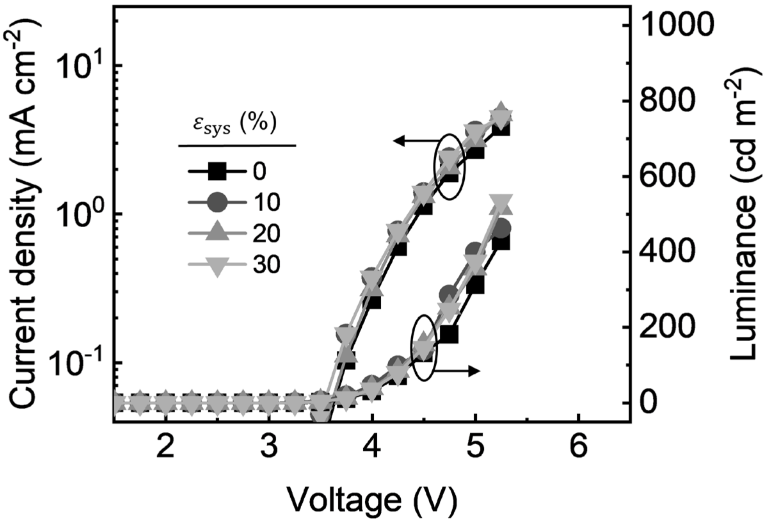

The difference in *J-V-L* characteristics in the high voltage region in **Fig. 5** is not only mechanical in nature but also stems from electrical degradation, which could be significant, especially when the maximum applied voltage exceeds a certain threshold. In experiments conducted with another sample from the same batch, sweeping under conditions less harsh than the maximum bias of 6V resulted in a lower degree of variation in the *J-V-L* characteristics among those with system strains of 30, 20, 10, and 0%, as depicted in above data.

Supplementary Figure 10 | The current efficiency (η_{CE}) characteristic versus luminance for the different biaxial system strain (ε_{sys}). (Inset: the black dot indicates the measurement spot where *L* was measured using CS 2000 (Konica-Minolta))

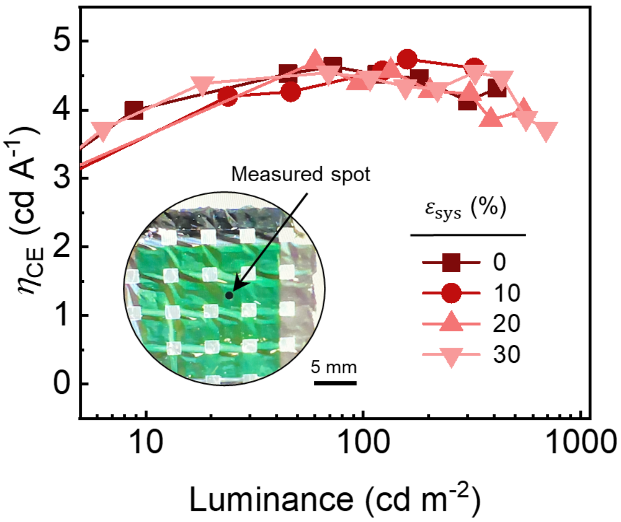

Supplementary Figure 11 | Analysis of optical characteristics in the HAA section. (a) Schematic diagram illustrating the light ray paths within the HAA in the initial and stretched states. (b) The distribution of angular spectra of the reference OLED having the same structure as used in the device fabrication. (Inset: the photograph of the reference OLED) (c) The angle-dependent change in CIE *x*, *y* coordinates of the reference OLED.

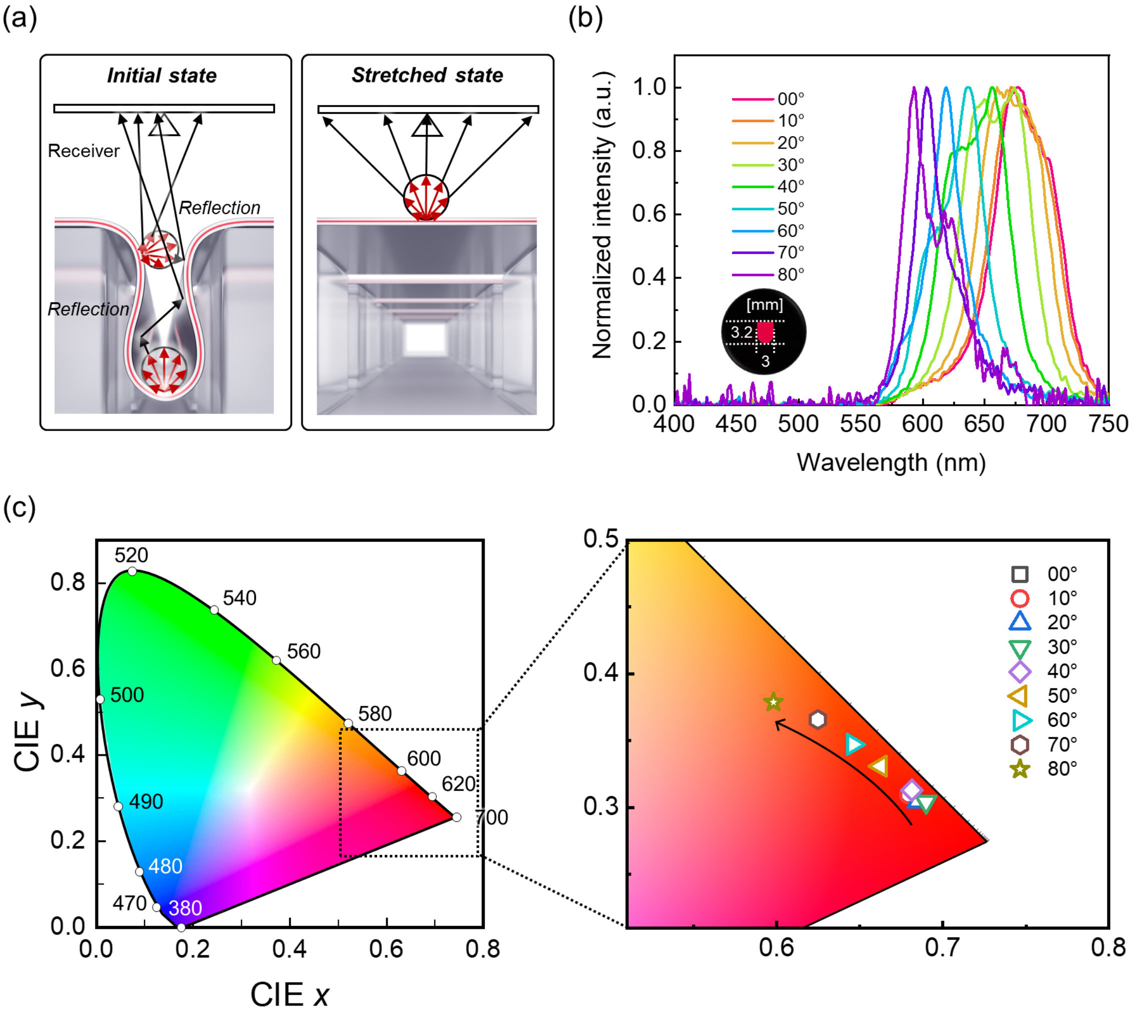

Supplementary Figure 12 | Measuring the optical characteristics of the entire surface area of the stretchable OLED according to the system strain. (a) Setup for measuring the CIE color coordinates of the entire OLED surface area using CA 2000 (Konica-Minolta) (b) Contour plots showing the color coordinates of the stretchable OLED under each system strain.

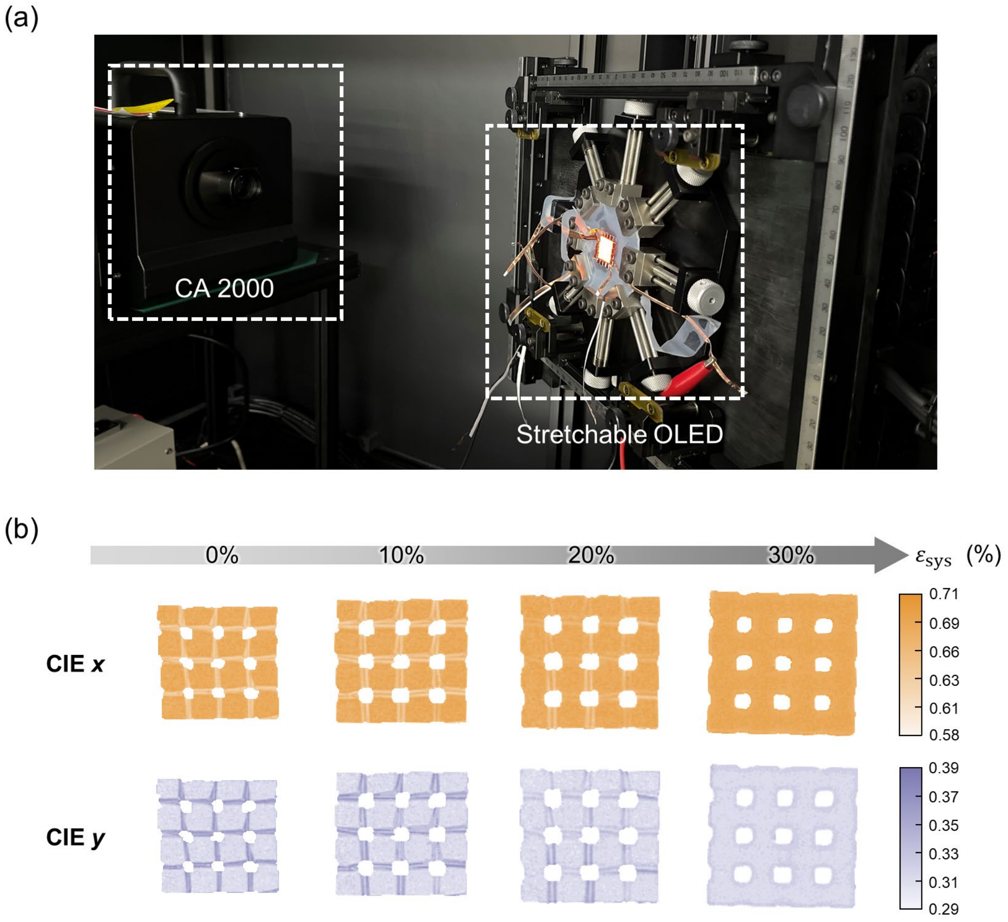

Supplementary Figure 13 | Biaxial cyclic test equipment set-up. (a) Schematic diagram of the set-up consisting of four clamps, two motors and an OLED driving module. (b) A photograph of the set up (inset: Loaded stretchable OLED with four clamping sites and a microscope).

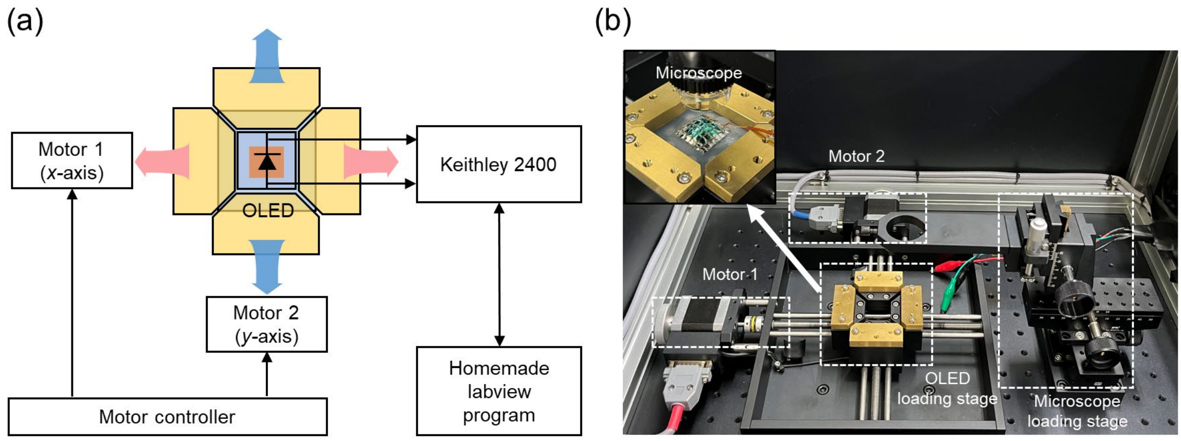

Supplementary Figure 14 | Observation of the durability of the stretchable OLED before and after 1000 stretch-release cycles. Photographs of the stretchable OLED devices driving with 1 mA (a) before and (b) after 1000 stretch-release cycles. (c) Lifetime test of stretchable OLEDs before and after 1000 stretch-release cycles. The result for a rigid reference device is also shown for comparison. (Under 100 nit driving condition, LT50 ≈ 6 hours in all cases. The inset shows photographs of the reference OLED operated at 100 cd m^{-2})

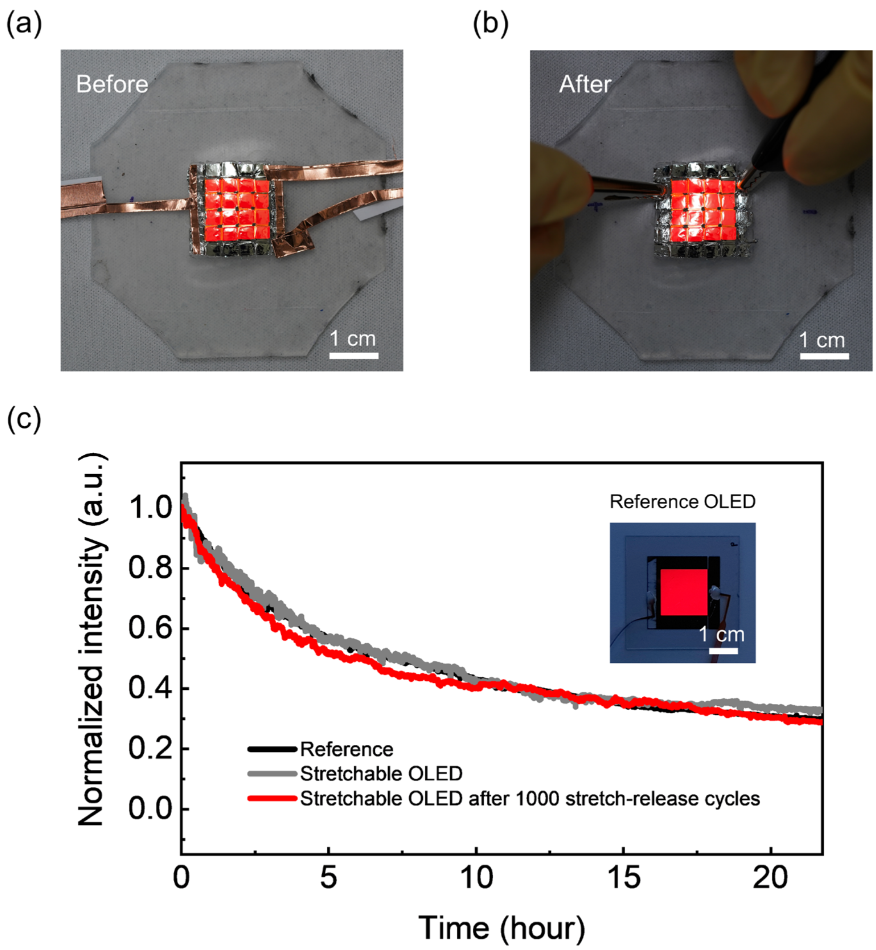

Supplementary Figure 15 | Demonstrations on various human body surfaces. (a) Back of hand, (b) elbow, and (c) arbitrary biaxial stretching using four hands.

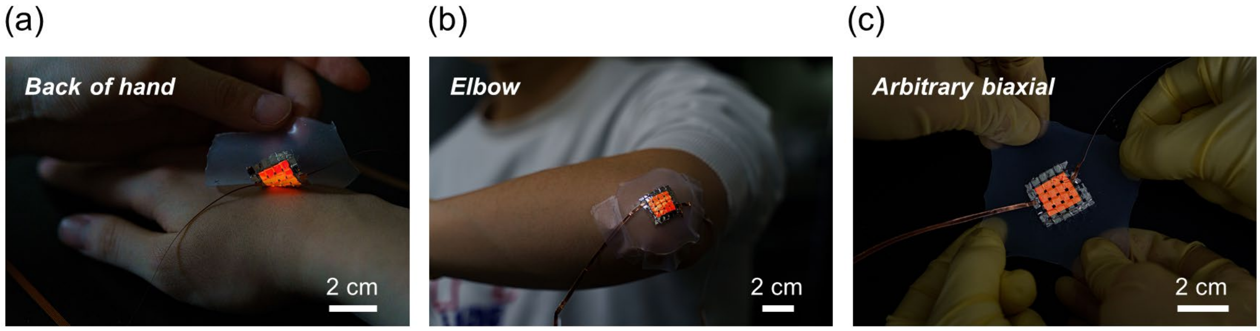

Supplementary Figure 16 | Strain analysis simulation under uniaxial stretching. (a) The ANSYS simulation results for y-axis deformation of the patterned-hybrid elastomer under uniaxial stretching with a 10% substrate strain. ($\varepsilon_{\mathrm{sub}} = 10\%$) in x-direction. Due to the substrate's Poisson's ratio, the island array is compressed perpendicular to the stretching direction, causing the distance between the islands to be approximately 0.2 mm closer compared to the initial state. As the distance between the islands decreases to less than 0.2 mm, different parts of the HAA can adhere to each other, limiting further stretching in the uniaxial direction. (b) The COMSOL simulation result illustrating the geometrical change along y-direction before and after applying $\varepsilon_{\mathrm{sub}} = 10\%$ in the x-direction. The vertical cross-section is shown for the section indicated as $\overline{PP'}$ in (a). (c) The simulated equivalent strain of the bottom Al_2O_3 and top Al_2O_3 are depicted along the x-axis for both the initial and compressed cases.

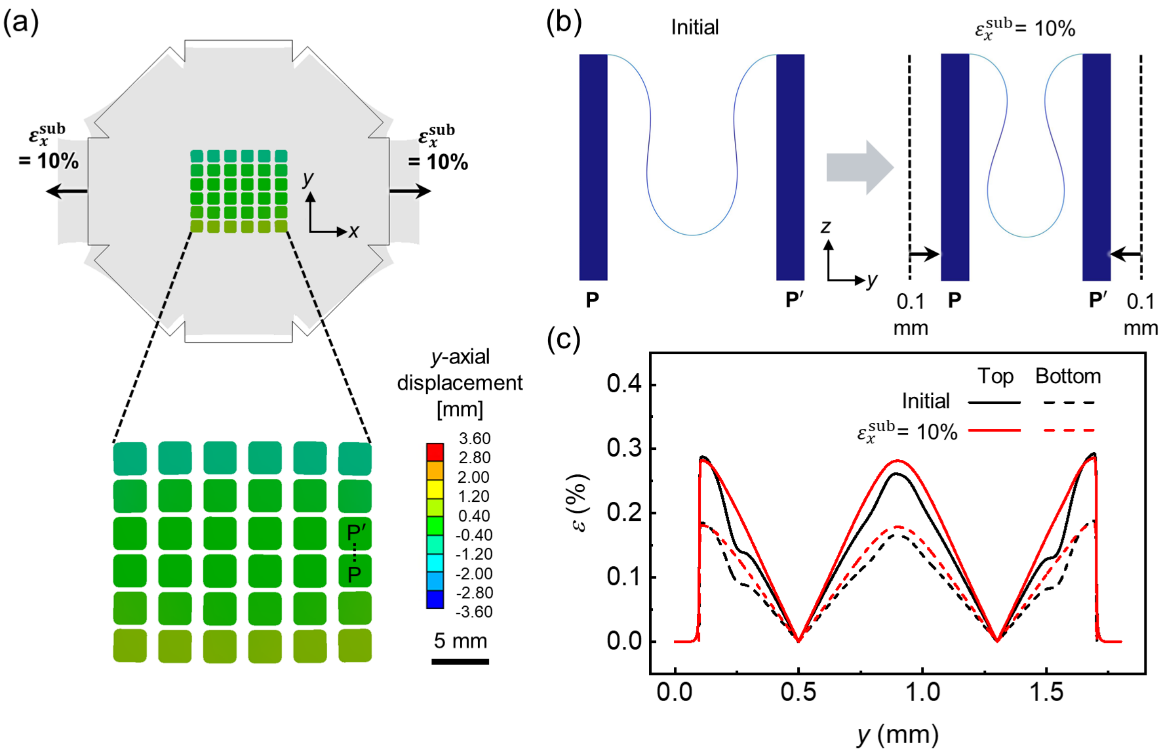

Supplementary Figure 17 | System setup for demonstration of a stretchable passive matrix (PM) display: (a) Schematic diagram and (b) photograph of the system setup.

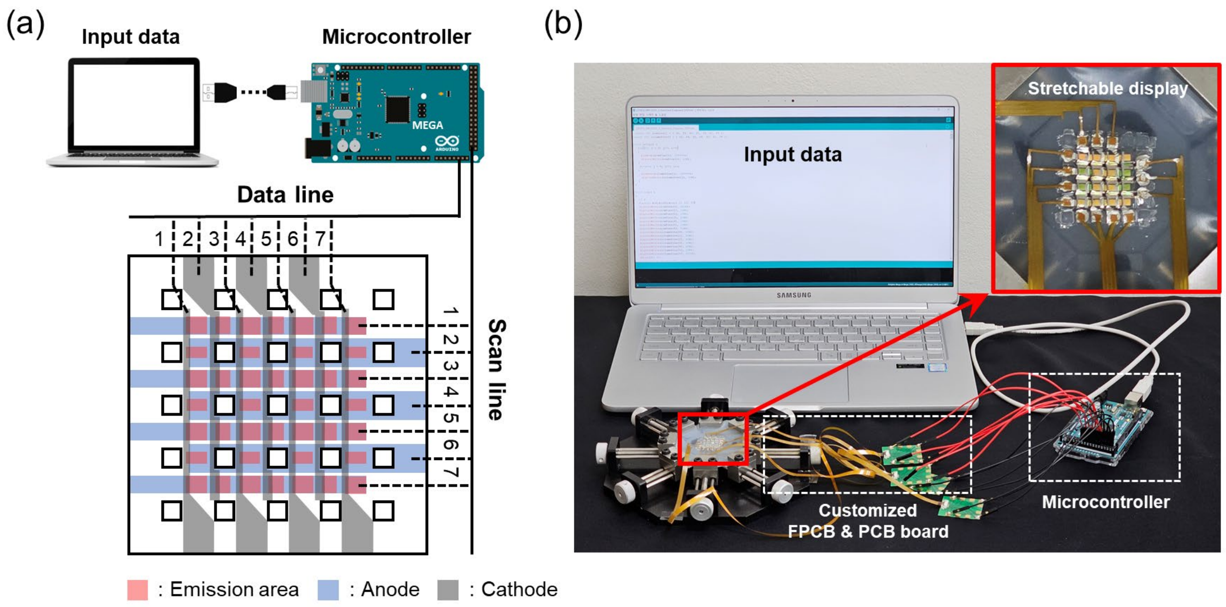

Supplementary Figure 18 | Shadow masks for PM stretchable OLED deposition. (a) Schematic diagrams of the guide mask and shadow masks defining the patterns of each layers in the proposed stretchable PM OLED device. The guide mask is used to securely hold the shadow masks for tight control of alignment margin. (b) Photograph of the guide mask and shadow masks.

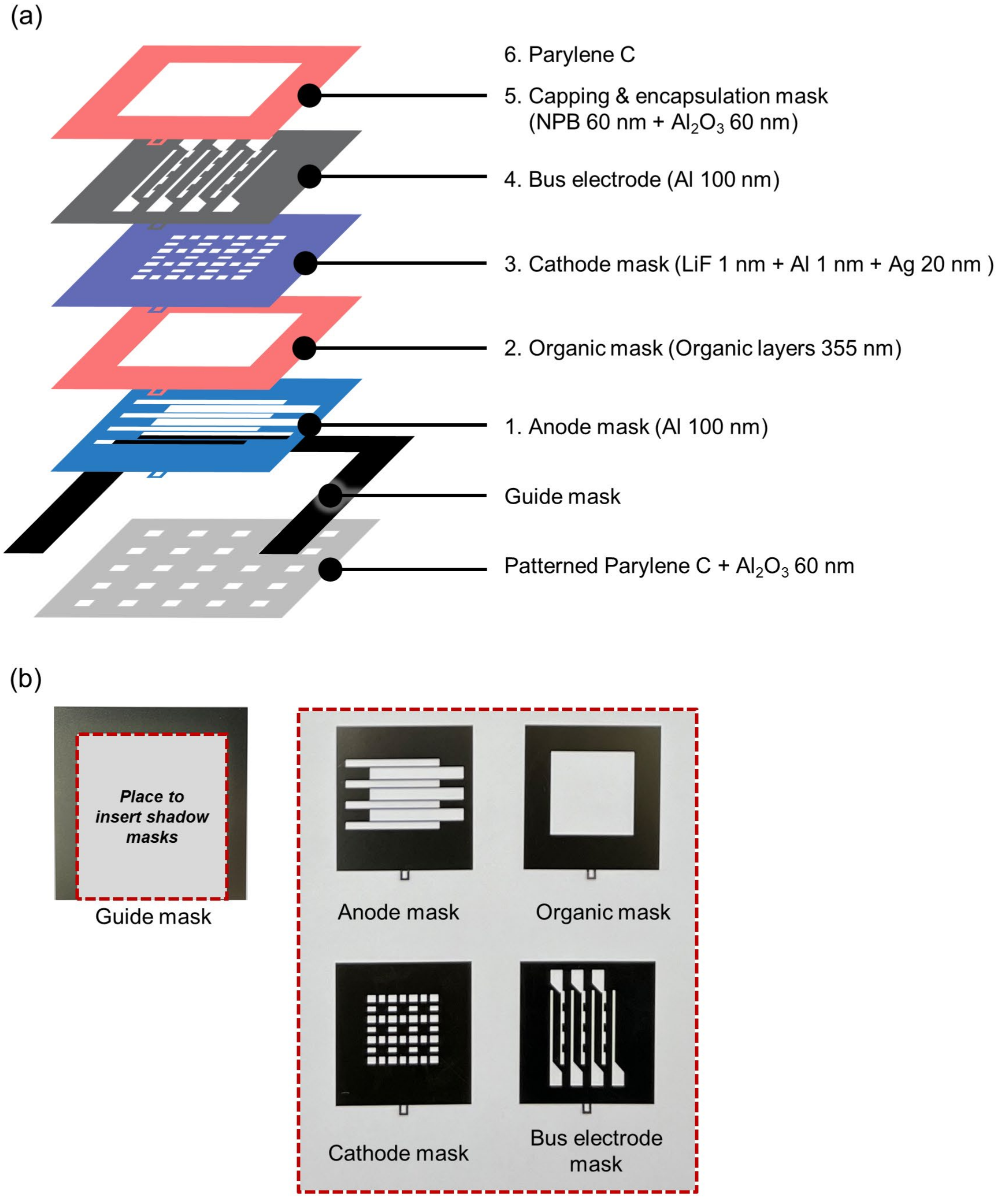

Supplementary Figure 19 | The top views of a deposited area through the shadow masks for each of the layers in OLEDs: (a) anode, (b) organic, (c) cathode, (d) bus electrode, and (e) capping and encapsulation layers. (f) Photograph of the fabricated stretchable PM OLED. Note that the substrate in (a)-(e) is the ultrathin parylene before integration onto the 3D patterned elastomer. The photograph in (f) is for the sample integrated onto the patterned elastomer. It is in a stretched state.

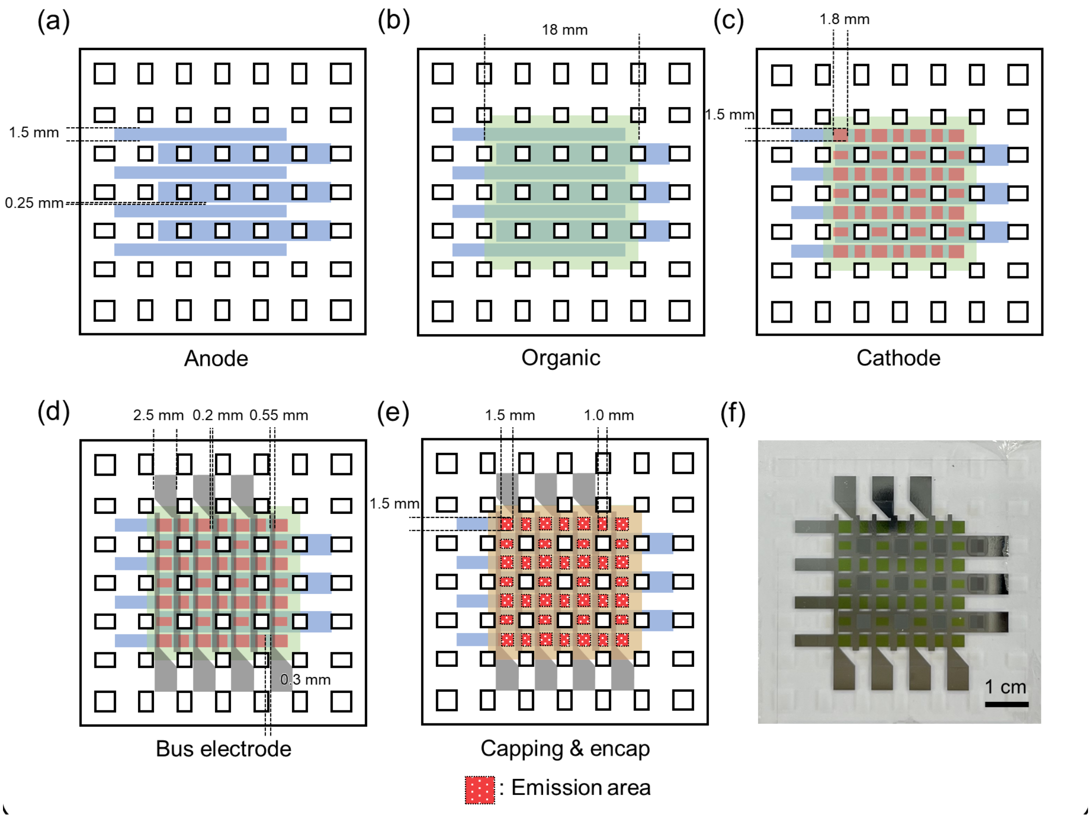

Supplementary Figure 20 | Concept diagram of PM demonstration utilizing hidden pixels. The conceptual diagrams depicting the 'D', 'I', 'S', 'P', 'L', 'A', and 'Y' alphabets through PM stretchable OLEDs: conventional devices in (a) the initial and (b) the stretched state without hidden pixels; the proposed devices with hidden pixels in (c) the initial and (d) the stretched state.

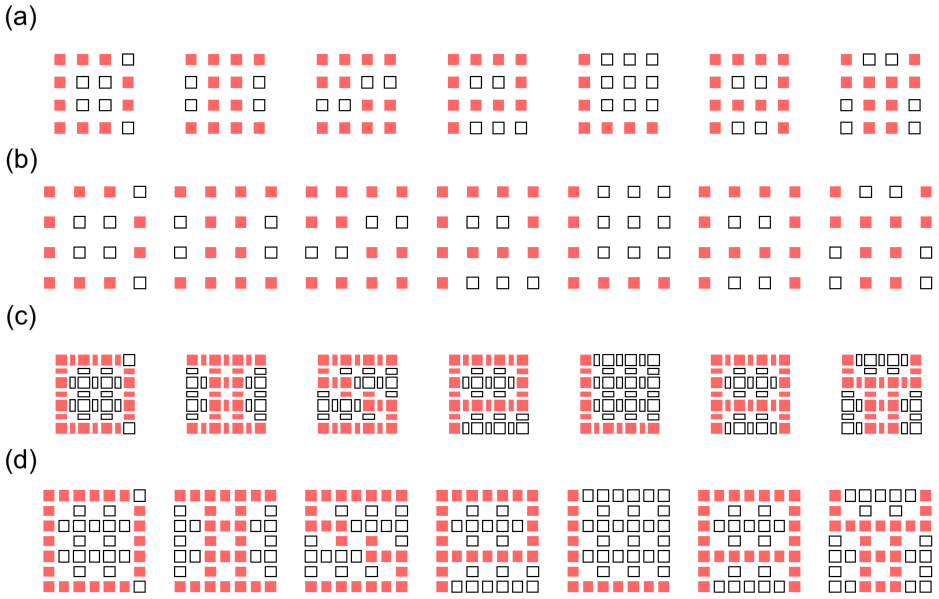

Supplementary Figure 21 | Schematic illustration and timing diagram of PM operation for seven alphabets. (a) The case mimicking the conventional PM operation without hidden pixels (4 × 4 operation) and (b) The proposed case with hidden pixels. (7 × 7 operation for resolution compensation)

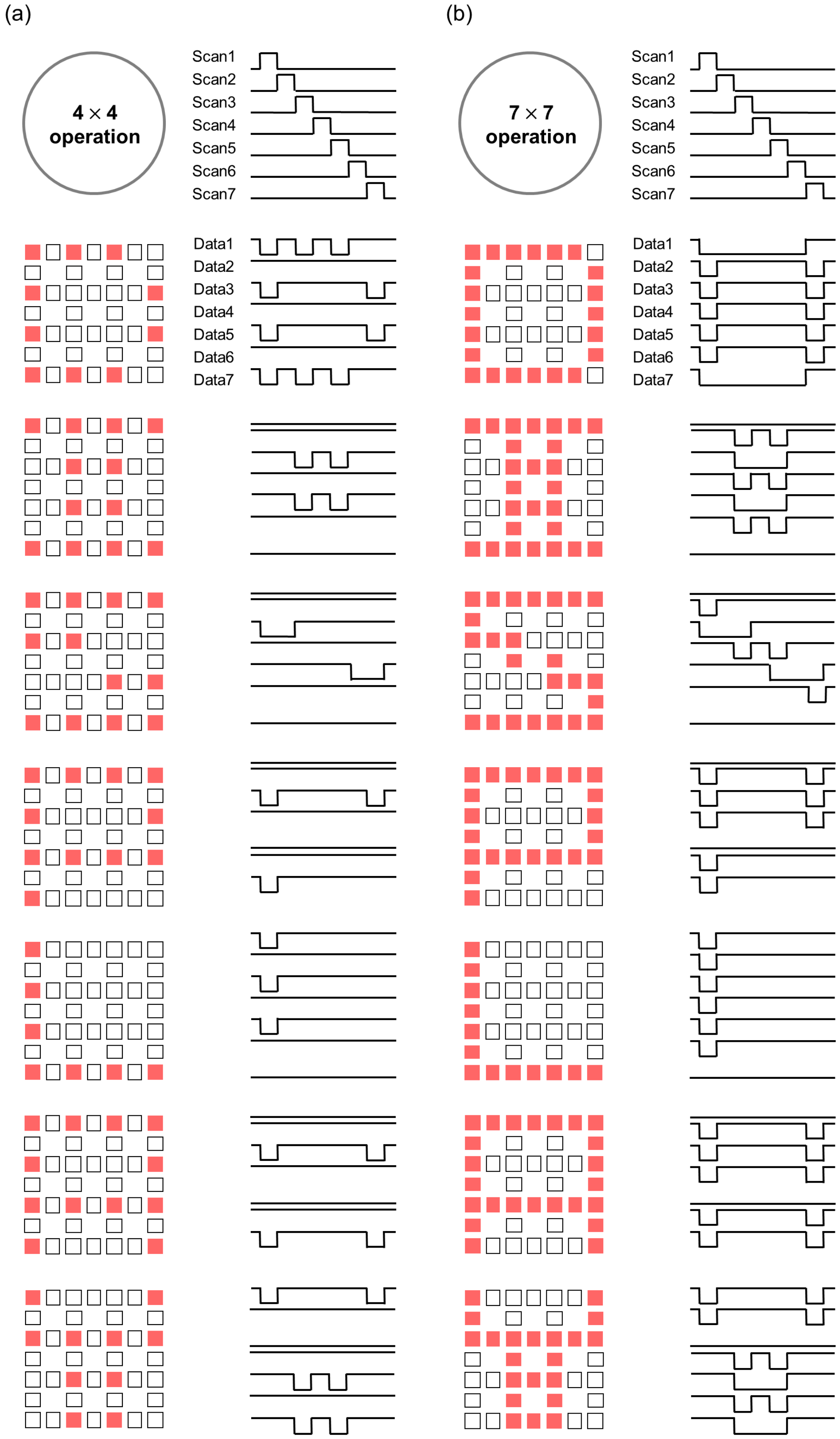

Supplement: Supplementary file 1 — Supplementary Information [file 41467_2024_48396_MOESM1_ESM.pdf]
