# Supplementary material for: Stretchable OLEDs based on a hidden active area for high fill factor and resolution compensation
Source: Nat Commun. 2024 Jun 4;15:4349. doi: 10.1038/s41467-024-48396-w (PMC11150391; doi:10.1038/s41467-024-48396-w)
Supplement: Supplementary file 3 — Description of Additional Supplementary Files [file 41467_2024_48396_MOESM3_ESM.pdf]

Description of Additional Supplementary Files

File name: Supplementary Movie 1
Description: 100 cycles of biaxial cyclic testing for the stretchable OLED (operated at 1 mA, 6× playback speed).

File name: Supplementary Movie 2
Description: Stretchable OLED adhered to an inflating balloon surface and stretched (operated at 1 mA, 3× playback speed).

File name: Supplementary Movie 3
Description: Low fill factor stretchable PM OLED display in the initial state without hidden pixels (operated at 5V, 2× playback speed).

File name: Supplementary Movie 4
Description: Low fill factor stretchable PM OLED display in the stretched state without hidden pixels (operated at 5V, 2× playback speed).

File name: Supplementary Movie 5
Description: High fill factor stretchable PM OLED display in the initial state with hidden pixels (operated at 5V, 2× playback speed).

File name: Supplementary Movie 6
Description: High fill factor stretchable PM OLED display with hidden pixels in stretched state (operated at 5V, 2× playback speed).

File name: Supplementary Movie 7
Description: A high fill factor stretchable PM OLED display operated at 10 Hz. The screen represents a waveform, with the x-axis denoting time and the *y*-axis representing the count of flickering. (For instance, in the case of 10 Hz, the *y*-value is set to 10 when the *x*-axis is at 1 second. The count resets to zero every one second) The white box outlined on the right side represents the enlarged PM OLED display.

File name: Supplementary Movie 8
Description: A high fill factor stretchable PM OLED display operated at 100 Hz. The screen represents a waveform, with the x-axis denoting time and the y-axis representing the count of flickering. The white box outlined on the right side represents the enlarged PM OLED display.
